# A Study on the Quality and Identity of Brazilian Pampa Biome Honey: Evidences for Its Beneficial Effects against Oxidative Stress and Hyperglycemia

**DOI:** 10.1155/2014/470214

**Published:** 2014-09-28

**Authors:** L. C. Cruz, J. E. S. Batista, A. P. P. Zemolin, M. E. M. Nunes, D. B. Lippert, L. F. F. Royes, F. A. Soares, A. B. Pereira, T. Posser, J. L. Franco

**Affiliations:** ^1^Interdisciplinary Center for Biotechnology Research, CIPBIOTEC, Universidade Federal do Pampa, Campus São Gabriel, 97300-000 São Gabriel, RS, Brazil; ^2^Department of Chemistry, Universidade Federal de Santa Maria, 97105-900 Santa Maria, RS, Brazil

## Abstract

We characterized, for the first time, the quality and identity of Brazilian Pampa biome honey and its antioxidant properties *in vitro* (FRAP, DDPH and ABTS). The potential protective effect of honey against oxidative stress induced by iron (Fe) and paraquat, (PQ) in a *Drosophila melanogaster* model (*in vivo*) was also tested. The results indicated that all honey samples tested showed antioxidant activity *in vitro*. Flies treated with honey showed increased lifespan and were protected against oxidative stress induced by Fe and PQ. Despite the high concentration of sugars in honey (approximately 70–80%), our results demonstrate a hypoglycemic-like effect of honey in *Drosophila*. Thus, this study demonstrates the high quality of Brazilian Pampa biome honey as well as its significant antioxidant activity *in vitro* and *in vivo*, pointing to the potential use of this natural product as an alternative in the therapy of oxidative stress-associated diseases.

## 1. Introduction

Honey is a complex mixture produced by honeybees from the nectar and is widely consumed as a sweetener. In addition, honey is well known for its therapeutic properties [1]. Honey is constituted primarily by sugars such as monosaccharides and disaccharide as well as organic acids, vitamins, carotenoid-derived compounds, amino acids, proteins, trace elements, phenolic compounds, and flavonoids. It also contains enzymes such as glucose oxidase, diastase, invertase, catalase, and peroxidase [2]. The composition of each honey is influenced by a number of factors including geographical origin, botanical sources of nectar, and environmental and climatic conditions as well as handling and processing techniques [3]. In addition, honey phenolic content has been correlated with the antioxidant capacity of honey samples from different regions [4, 5]. Thus, it becomes important to characterize the identity and quality of honeys from distinct regions around the globe.

The Brazilian Pampa biome is one of the six biomes occurring in Brasil and covers a large area shared with Argentina and Uruguay in the southern portion of South America. It presents distinctive characteristics of vegetation, climate, and soil types, making it a unique ecosystem on the planet [6]. Brazil is one of the largest honey producing countries, exporting this natural product to almost all continents, including Europe. The Rio Grande do Sul state (where the Pampa biome is located) is the largest honey producer in Brazil [7]. But so far, no studies on the characterization of honeys from the Pampa biome are available.

It is shown from previous reports that honey can deliver health benefits such as antihypertensive, reproductive, anti-inflammatory, antifungal, antibacterial [8], hypoglycemic [9, 10], and antioxidant effects [1, 10]. In many of these studies, researchers commonly use rodent based models such as rats and mice [8]. However, there is no registry of the use of alternative animals in studies of honey quality and characterization of its biological properties.

The fruit fly,* Drosophila melanogaster*, is a model-organism that has been used due to the advantages arising from its life cycle, as the rapid development and easy handling making them ideal organisms for the use on* in vivo* bioassays [11, 12]. Although humans and* D. melanogaster* are only distantly evolutionarily related, almost 75% of disease-related genes in humans have functional orthologs in the fly, making it a reasonable model system for human diseases.* Drosophila* have been also proving to be a powerful model system for the study the development and functioning of nervous system and for the study of fundamental cellular pathways responsible for metal and insecticide toxicity [12].

Recent studies have shown that natural products and isolated polyphenols have antioxidant activity and are able to block oxidative stress induced by redox-active chemicals in* Drosophila* models of human diseases, including Parkinson's disease [11, 13]. However, to date, there are no studies regarding the beneficial effects of honey in these models.

In this way, considering the lack of studies on the quality and identity of the Brazilian Pampa biome honey, as well as its therapeutic potential, it is relevant to characterize the physicochemical parameters of this natural product as well as evaluating its antioxidant/protective properties. Thus, in the present study we focused on the characterization of Brazilian Pampa biome honey, as well as its antioxidant properties* in vitro* and* in vivo*, using an iron/paraquat model of oxidative stress in* Drosophila melanogaster*, thus, developing an alternative model for studying the antioxidant and protective effects of honey.

## 2. Material and Methods

### 2.1. Chemicals

Sodium bisulfate, copper (ii) sulfate, potassium sodium tartrate tetrahydrate, tannic acid, Folin-Ciocalteu, 2,2′-azino-bis(3-ethylbenzothiazoline-6-sulfonic acid) diammonium salt, sodium acetate, HEPES, minimum 99.5% titration, 2,4,6-tris (2-pyridyl)-5-triazine (TPTZ), methyl viologen dichloride hydrate (Paraquat), amyloglucosidase from* Aspergillus niger*, and 2,2-di(4-tert-octylphenyl)-1-picrylhydrazyl free radical (DPPH) were purchased from Sigma-Aldrich (São Paulo, SP, Brazil). Glucose PAP liquiform (Kit) was purchased from Labtest (Belo Horizonte, MG, BRA) and potassium ferrocyanide trihydrate, zinc acetate dihydrate, iron(II) ammonium sulfate hexahydrate, gallic acid monohydrate, aluminum chloride hexahydrate, potassium persulfate, and iron(III) chloride were purchased from Vetec Química Fina LTDA (Rio de Janeiro, RJ, BRA). All the other chemicals used in this work were of analytical grade.

### 2.2. Honey Sampling

**Figure 1:**
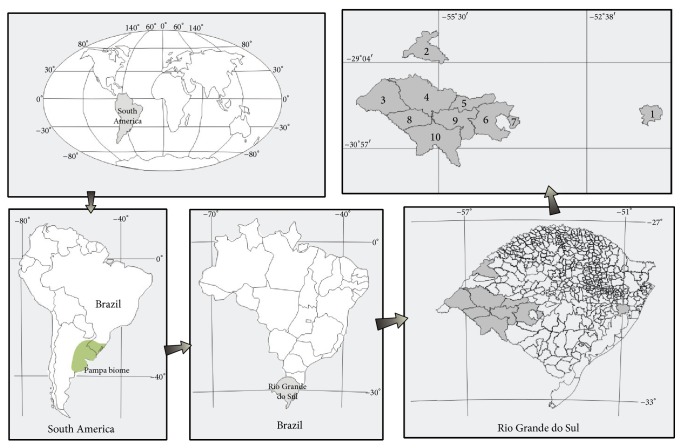
Brazilian Pampa biome representative map showing the locations of honey sample collection. 1: Viamão; 2: São Borja; 3: Uruguaiana; 4: Alegrete; 5: Cacequi; 6: São Gabriel; 7: Vila Nova; 8: Quaraí; 9: Rosário Do Sul; 10: Santana Do Livramento.

We used ten (*n* = 10) honey samples from* Apis mellifera* from different areas of the Brazilian Pampa biome (Figure 1) and they were identified according to floral origin and harvest date (Table 1). The samples were provided by the Cooperativa Apícola do Pampa Gaúcho LTDA (COAPAMPA), located in the city of São Gabriel in the state of Rio Grande do Sul, Brazil. All honey samples were stored at room temperature (20–25°C) in plastic containers until analysis.

### 2.3. Physicochemical Analysis

The methods used for determination of quality parameters were according to Brazilian regulation, which determines the technical procedures for attachment of identity and quality of honey [14], following international standards. The methods advocated by this legislation follow the* Codex Alimentarius* Commission [15] and the Association of Official Analytical Chemists [16]. Qualitative analysis, according to that described by Instituto Adolfo Lutz [17], was also made to determine possible adulteration of honeys, complementing those provided by the Brazilian law.

#### 2.3.1. Moisture

Moisture was determined using refractometric method of Chataway [16]. All measurements were taken using an Abbe refractometer (Carl Zeiss, Germany) with honey at 20°C. The value of refractive index indicated was converted to the moisture content using a reference table (Table Chataway), which provides the percentage of moisture content in honey samples.

#### 2.3.2. Free Acidity and pH

Honey was diluted with distilled water and pH was measured using a pH meter. Then, the free acidity of the sample was determined by titration with NaOH. The total volume required in titration procedure was used for the calculation of the free acidity, represented as milliequivalents per kg of honey [16].

#### 2.3.3. Water Insoluble Solids

We used a gravimetric method in which honey was diluted with the least possible water at temperature of 80°C. The solution was filtered using a glass crucible (pore 15.40 *μ*M) until free sugar was left. The crucible was dried at 135°C for one hour. The insoluble solids concentration is calculated from the weight difference before and after the crucible honey passage and expressed in percent of insoluble solids present in sample honey [15].

#### 2.3.4. Hydroxymethylfurfural (HMF)

HMF was determined by the method described by White Jr. [18]. Honey was diluted in water and both Carrez I (15% potassium ferrocyanide) and Carrez II (30% zinc acetate) reagents were added. After filtration, a reference sample was obtained by addition of 0.2% (w/v) sodium bisulfite. Absorbance was determined at 284 and 336 nm in a quartz cuvette in a Thermo Scientific Evolution 60s UV-Visible spectrophotometer. HMF contents are expressed as mg/kg.

#### 2.3.5. Reducing Sugars and Apparent Sucrose

The determination of reducing sugars and apparent sucrose was conducted by titration in which the colorimetric change of the reaction indicated the percentage concentration of reducing sugars in the sample [15]. The reaction occurs with the honey sample (0.5%) as titrant, which reduces copper (blue color) at boiling point of Fehling's reagent (44.21 mg/mL copper sulphate and 346 mg/mL sodium potassium tartrate + 100 mg/mL sodium hydroxide) in cuprous oxide (red color) using 0.2% methylene blue as indicator. The apparent sucrose content was determined after inversion by acid hydrolysis (1 mL hydrochloric acid) and the value subtracted from the value obtained for total reducing sugars.

#### 2.3.6. Lugol's Reaction

Lugol's reagent is composed of iodine (20 mg/mL) and potassium iodide (60 mg/mL). 10 g honey was dissolved in 20 mL of distilled water and kept in a water bath for 1 h. Then, an aliquot of 0.5 mL Lugol's reagent was added to the solution. This is a qualitative test used to investigate the presence of starch and dextrin in honey. It is considered positive when the final color varies from violet to blue, indicating adulteration [17].

#### 2.3.7. Lund's Reaction

Two grams of each sample was weighted and diluted in 20 mL of distilled water in a graduated cylinder. Then, 5 mL of tannic acid (0.5%) was added to the diluted honey. The final volume of each sample was adjusted to 40 mL with distilled water. The mixture was shaken and allowed to rest for 24 h. In the presence of pure honey an albuminoid precipitate (0.6–3.0 mL) is developed [17].

#### 2.3.8. Total Protein

The total protein content in honey (0.1 g/mL) was estimated as described by Bradford [19] using bovine serum albumin (BSA) as standard. The absorbance was measured at 595 nm with the aid of an EnSpire multimode plate reader (PerkinElmer, USA). Total protein contents present in honey sample is expressed as mg/g.

### 2.4. Analysis of Antioxidant Properties

All the spectrophotometric assay of the analysis of antioxidant properties was performed in 96-well plates using the EnSpire multimode plate reader (PerkinElmer, USA).

#### 2.4.1. Total Phenolics

Phenolic compounds from honey samples were detected by the Folin-Ciocalteu method [20] with minor modifications. Briefly, 4 *μ*L honey solution (0.1 g/mL) was mixed with 35 *μ*L 1 N Folin-Ciocalteu's reagent. After 3 min, 70 *μ*L 15% Na_2_CO_3_ solution was added to the mixture and adjusted to 284 *μ*L with distilled water. The reaction was kept in the dark for 2 h, after which the absorbance was read at 760 nm. Gallic acid was used as standard (10–300 *μ*g/mL). The results were expressed as mg of gallic acid equivalents (GAEs) per 100 g honey.

#### 2.4.2. Flavonoid Content

The total flavonoid content was determined using the Dowd method as adapted by Arvouet-Grand et al. [21]. Briefly, 150 *μ*L of 2% aluminium trichloride (AlCl_3_) was mixed with the same volume of a honey solution (0.01 g/mL). The blank consisted of 150 *μ*L honey solution with 150 *μ*L methanol without AlCl_3_ and after 10 min the absorbance was read at 415 nm. Quercetin was used as standard (0–25 *μ*g/mL). The results were expressed as mg of quercetin equivalents (QE) per 100 g honey.

#### 2.4.3. DPPH^∙^ Radical Scavenging Assay

The scavenging activity towards 2,2-diphenyl-1-picrylhydrazyl (DPPH^∙^) radical was evaluated according to the method of Baltrušaitytė et al. [22] with minor modifications. In brief, 30 *μ*L of DPPH^∙^ (900 *μ*M) diluted in methanol was mixed with 50 *μ*L of honey (0.1 g/mL) in a 96 wells microtitre plate. The final volume of each well was adjusted to 300 *μ*L with methanol. Ascorbic acid was used as a positive control. The absorbance was determined at 517 nm after 45 min incubation. The radical scavenging activity (RSA) was calculated by the formula: %RSA = [(AB − AA)/AB] × 100, where RSA = DPPH^∙^ scavenging; AB = absorption of a blank sample (only DPPH^∙^); AA = absorption of a tested honey sample.

#### 2.4.4. ABTS^∙+^ Radical Scavenging Assay

The antioxidant activity of honey sample in the reaction with ABTS^∙+^ radical was determined according to the method of Baltrušaitytė et al. [22] with some modifications. ABTS^∙+^ radical solution was generated by oxidation of solutions prepared of 1 mL of 7 mM 2,2′-azino-bis(3-ethylbenzothiazoline-6-sulfonic acid) diammonium salt stock solution with 17.5 *μ*L of 140 mM potassium persulfate (K_2_S_2_O_8_). 200 *μ*L of ABTS^∙+^ solution was mixed with 10 *μ*L of honey solution (0.1 g/mL) in a microplate and the decrease in the absorbance was measured after 10 min. Ascorbic acid (1 mM) was used as a positive control. The radical scavenging activity (RSA) was calculated by the formula of %RSA, the same described for DPPH^∙^ radical.

#### 2.4.5. Ferric Reducing Antioxidant Power (FRAP)

The reducing capacity of honey samples was assayed with the original method of Benzie and Strain [23], adjusted to analysis of honey samples. 9 *μ*L of honey sample (0.1 g/mL) was mixed with of 270 *μ*L of freshly prepared FRAP reagent. The FRAP reagent was prepared by mixing 2.5 mL of 0.3 M acetate buffer pH 3.6 with 250 *μ*L of 10 mM 2,4,6-Tris(2-pyridyl)-s-triazine (TPTZ) solution and 250 *μ*L of FeCl_3_·6H_2_O. The mixture was shaken and left in a water bath for 30 min and the absorbance readings were taken at 595 nm. Ammonium iron(II) sulfate hexahydrate was used to calculate the standard curve (100–2000 *μ*M). The reducing ability of honey was expressed as *μ*M of Fe(II) equivalent/100 g honey.

#### 2.4.6. Color Intensity (ABS_450_)

Color intensity was determined by the method of Piljac-Žegarac et al. [24]. Honey was diluted to 50% (w/v) in warmed (45–50°C) milli-Q water and filtered (0.45 *μ*m pore size) to remove large particles. The absorbance was measured in plate reader at 450 and 720 nm, and the difference in absorbance was expressed as mAU.

### 2.5. *Drosophila* Stock

*D. melanogaster* (Harwich strain) was obtained from the National Species Stock Center, Bowling Green, OH. The flies were reared in glass vials containing Bloomington standard cornmeal* Drosophila* medium [12] in a constant temperature and humidity (20°C ± 1; 60% relative humidity). All experiments were performed with the same strain, using adult flies at 0–4 days old.

### 2.6. *D. melanogaster* Treatments

#### 2.6.1. Paraquat and Iron Exposure

**Figure 2:**
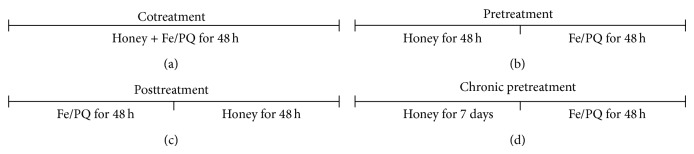
Schematic representation of* Drosophila melanogaster *treatment schedule. (a) Cotreatment with honey and Fe or PQ during 48 h; ((b) and (d)) pretreatment with honey during 48 h (b) or 7 days, (d) respectively, following Fe or PQ exposure for 48 h; (c) posttreatment with honey for 48 h after Fe or PQ exposure for 48 h.

In order to check for resistance to oxidative stress, flies were exposed to iron (Fe; 10 or 15 mM) and paraquat (PQ; 10 or 20 mM), based on the methodology described by Jimenez-Del-Rio et al. [11]. Different treatments were performed, but with the same purpose (Figure 2). In a first treatment schedule, flies were acutely exposed to Fe and PQ in the presence or absence of honey for 48 h. In a second treatment schedule, 20 flies per group (*n* = 6) were administered with honey then exposed to prooxidants for 48 h.

#### 2.6.2. Lifespan

For lifespan experiments, 10 sets of 20 male flies were assayed for each experimental group (*n* = 10). In the control group flies were kept in standard medium; the honey group was treated with 10% honey (w/w) in standard medium and the glucose + sucrose group received equivalent amounts of sugars (as to the amount present in 10% honey) in standard medium. The honey used for* Drosophila* treatment contained 76.8 ± 0.9% reducing sugars and 3.0 ± 0.5% sucrose. The flies were evaluated during 45 days, in which the food was replaced every 10 days. The percentage of survival was estimated every five days.

#### 2.6.3. Total Glucose and Glycogen

Ten females flies from each group: control, 1% sucrose; honey, 10% solution; glucose + sucrose were treated for 7 days on filter paper, replacing the solutions every 24 h. Flies were anesthetized in ice, weighed (to reach 10 mg of flies) and transferred to chilled microcentrifuge tubes containing 1 mL buffer 20 mM HEPES buffer (pH 7.0). The whole fly bodies were homogenized in a PowerLyzer 24 Bench Top Bead-Based Homogenizer (MOBIO, Carlsbad, CA, EUA). After centrifugation (20,000 ×g for 30 min.), 10 *μ*L of the supernatants was used for determination of glucose by using a glucose detection kit (Labtest) in a multimode plate reader (EnSpire-PerkinElmer, USA). Glycogen was determined by conversion into glucose after addition of 0.1 U/mL amyloglucosidase. Following incubation for 15 min at 60°C, glucose was determined and values were subtracted from total glucose [25, 26]. Results were expressed as percent of control.

### 2.7. Locomotor Assay

The locomotor deficits were evaluated by negative geotaxis assay according to Jimenez-Del-Rio et al. [11] with minor modifications. After treatments flies were transferred to test tubes marked at 5 cm height. The flies were gently tapped to the bottom of the tube and the number of flies able to climb 5 cm after 6 seconds was recorded at 1-minute intervals. Each experiment was repeated thrice. The climbing performance index (PI) was calculated according to the following equation: 1/2[(*n*_tot_ + *n*_top_ − *n*_bot_)/*n*_tot_], where *n*_top_ = numbers of flies at the top, *n*_bot_ = at the bottom, and *n*_tot_ = total number of flies. Results were expressed as percentage of control.

### 2.8. Statistical Analysis

Statistical analysis was performed by one-way ANOVA followed by Tukey's* post hoc* test, using the GraphPad Prism (version 5) software. Differences were considered to be significant at the *P* < 0.05 level.

## 3. Results and Discussion

### 3.1. Physicochemical Parameters

The results from the analysis of identity and quality parameters of Brazilian Pampa biome honeys are summarized in Table 2. In Table 3 are summarized standards international, preconized by the Codex Alimentarius Commission (CAC) and the Association of Official Analytical Chemists (AOAC) required for Brazilian honeys. In this regard, for a better understanding, moisture, reducing sugars, and apparent sucrose are quality parameters related to honey maturity; for example, immature honeys may present higher humidity, which can lead to undesirable fermentation. Insoluble solids are related to honey purity, while pH, free acidity, and HMF are related to honey deterioration. HMF, Lugol/Lund's reaction is also related to potential adulterations of honey [1, 27].

HMF is an aldehyde resulting from degradation of fructose in honey. The determination of its content is one of the most important indicators of honey quality. HMF is naturally produced during aging of the honey; however, its formation can be accelerated when adulterations such as overheating, addition of plain sugars, and/or acidification take place [28]. Lund's and Lugol's reaction are qualitative analysis to determine possible adulteration of honey. Lugol's reaction is based on the reaction between iodine and potassium iodide in the presence of commercial glucose, sugar syrups, or dextrin in honey, resulting in a stained solution (red-purple to blue). Lund's reaction is based on the precipitation of honey's proteins by the tannic acid. The reaction is considered positive, indicating the presence of pure honey, when the precipitate varies from 0.6 to 3.0 mL [27]. There is no any regulation or legislation imposing limits for the protein content and pH in honey, but it is known from literature that the honey is naturally acidic irrespective of its geographical origin, which may be due to the presence of organic acids that contribute to its stability against growth of microorganisms [29]. The protein levels can be attributed mainly to the presence of different types of enzymes and other products that were introduced by the bees from the pollen and flower nectar. Protein content in honey depends on their botanical or geographical origin and storage time [30]. According to Bogdanov et al. [2] the honey contains roughly 0.5% proteins.

The results of the 10 honey samples regarding quality parameters, shown in Table 2, demonstrated that all honeys tested are in accordance with the international official required limits (Table 3). Compared with other studies with honeys from other regions of Brazil, reported in literature, Brazilian Pampa biome honeys achieve better quality parameters [31–33]. Confirming for the first time that Brazilian Pampa biome honeys present an acceptable quality with respect to the physicochemical parameters tested, as well as an identity when compared to other Brazilian honeys. This quality can be related to the unique ecosystem characteristics found in the Pampa biome, as mentioned before.

### 3.2. Antioxidant Properties* In Vitro*

The antioxidant activity of honey is directly related to its chemical composition, especially to the presence/concentrations of phenolic compounds and flavonoids. The concentrations of these compounds vary depending on the floral origin of the honey samples [29, 30].  Table 4 summarizes Brazilian Pampa biome honey* in vitro* antioxidant properties. The total phenolic content ranged from 41.0 to 69.0 mg of GAE/100 g between samples. The flavonoids content varied from undetected (by the method used) to 4.2 mg of QE/100 g and the color intensity 120.5–490 mAU between samples. Comparing to other studies that employed the same method, the phenolic content of the honeys we analyzed is similar to the values found for Burkina Fasan [34], Croatian [24], and Mexican [1] honeys and higher levels than Algerian [29], Bangladeshi [30], and Malaysian [35] honeys. The flavonoid content also matches those found in literature reports [34]. It was also observed that light colored honeys presented a lower content of phenolic compounds when compared to darker ones. This can be explained by the fact that polyphenols contribute to the color of honeys [36]. This correlation between color and phenolic compounds also was observed in other studies [24, 29, 30]. Thus, our results indicate that honey samples from Brazilian Pampa biome are of equivalent, and, in some cases, of superior quality in terms of antioxidant properties when compared to honeys tested worldwide.

The ferric reducing antioxidant power (FRAP) of Pampa biome honeys was tested. The principle of this method is based on the reduction of complex Fe^3+^-TPTZ to the form Fe^2+^-TPTZ in the presence of antioxidants [30]. The results on Table 4 summarize FRAP values obtained for the tested honeys. The results ranged from 133.0 to 537.2 0 *μ*M Fe[II]/100 g of honey. The FRAP values of the honey samples analyzed are similar to those found in literature [1, 24, 29, 30, 35]. In order to complement the evaluation of antioxidant activity of Brazilian Pampa biome honeys, the DPPH^∙^ and ABTS^∙+^ radical scavenging capacity were also tested. All honeys showed scavenging potential for both radicals. The percent scavenging capacity for DPPH^∙^ assay ranged from 20.9 to 44.2 between the honey samples and percentage scavenging of ABTS^∙+^ ranged from 14.0 to 34.3. The* in vitro* antioxidant activity found for Brazilian Pampa biome honeys is in the range of those found in literature [22, 37].

A positive correlation between the presence of phenolic compounds and honey color and the antioxidant capacity* in vitro* was observed (Table 5). A significant correlation (*P* < 0.05) among FRAP, ABTS^∙+^ radical scavenging, and DPPH^∙^ radical scavenging was found, however, a lower (*R* = 0.615) correlation between phenolic compounds and DPPH^∙^ radical scavenging test. This can be justified by the fact that DPPH^∙^ radical reacts mainly with lipophilic antioxidants while ABTS^∙+^ radical reacts with both hydrophilic and lipophilic antioxidants [24]. The color of Brazilian Pampa Biome honeys was positively correlated (*P* < 0.05) with phenolic compounds content and antioxidant capacity tests. This results show that honey's color can, at least in part, reflect the antioxidant capacity, as already observed elsewhere [36]. The lack of correlation between flavonoids content with color intensity, phenolic compounds, and consequently antioxidant capacity tests needs further elucidation; however, at least in part, it may be justified by the low sensitivity of the method used in this study. According of the Meda et al. [34], flavonoid content determined by the aluminum chloride method is specific only for flavones and flavonols. This means that this method alone underestimates the real content of total flavonoids presented in samples.

### 3.3. Lifespan

**Figure 3:**
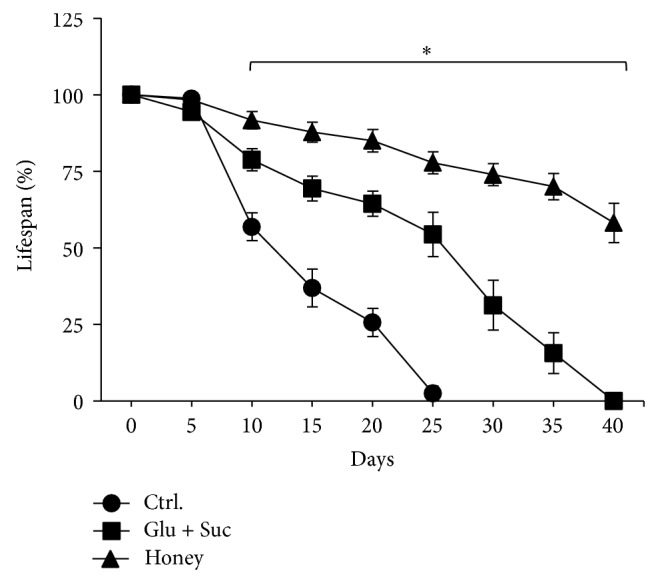
Effect of honey and sugars on* Drosophila melanogaster* lifespan. Flies were treated in standard medium over the course of the treatment schedule. The control group received standard medium only; honey group was administered a 10% honey solution (w/w) and the glucose + sucrose (glu + suc) group received equivalent amounts of sugars (as to the amount present in 10% honey). Lifespan was followed during 45 days. Statistically significant differences were observed between groups starting after the 10th day of treatment (*P* < 0.05).

The antioxidant capacity of several compounds and natural products has been correlated with their potential protective effects against oxidative stress-related comorbidities [10, 13, 37]. In order to test the potential beneficial effects of Brazilian Pampa biome honey, we evaluate the lifespan of flies treated with honey (10%) and their sugar equivalents in order to respond whether honey and or its sugars would prolong the lifespan of flies. The experiment lasted 45 days. As seen in Figure 3, honey significantly increased lifespan. Flies that were administered sugars, at the same amount as present in honey, had an increased lifespan when compared to control; however, they did not match to the honey treatment. This indicates that sugars present in honey may contribute in part for the observed prolongation of survivorship; however, additional nonsugar compounds may contribute as main factors by which Brazilian Pampa biome honey exerts its effects on flies lifespan. Studies have shown that high concentrations of sugars, due to its high energy content, decrease mortality of* Drosophila* [13]. However, the honey complex composition in which besides sugars also contains vitamins, amino acids, proteins (such as enzymes), phenolic compounds, flavonoids, and mineral salts, among others [2] ended up contributing with a greater survivability of treated flies.

### 3.4. Antioxidant Properties* In Vivo*: Paraquat and Iron Exposure

**Figure 4:**
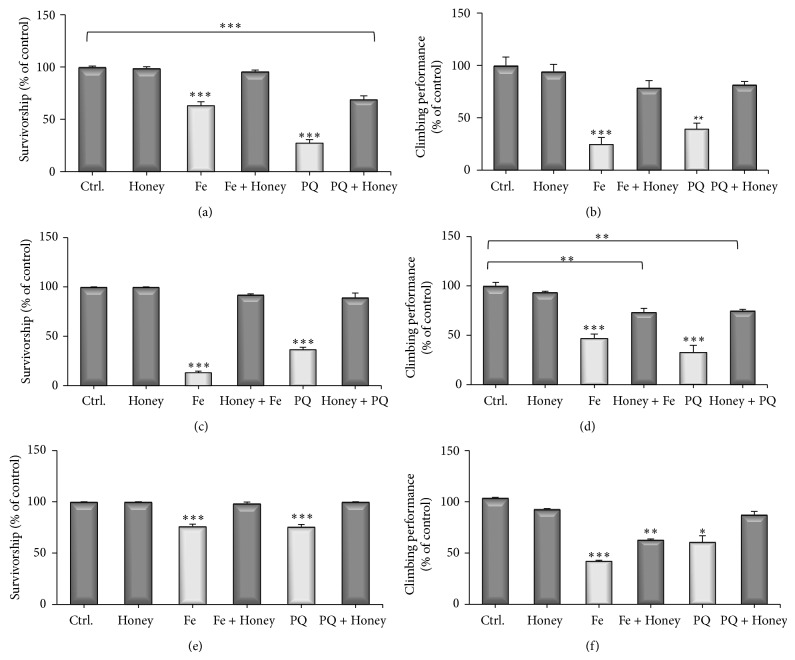
Flies treated with honey were protected against oxidative stress induced by Fe and PQ in different treatments. Cotreatment of flies with honey and Fe or PQ for 48 hours. (a) Percentage of surviving flies after treatment; (b) locomotor activity test. Pretreatment of flies with honey and Fe or PQ for 48 hours. (c) Percentage of surviving flies after treatment; (d) locomotor activity test. Posttreatment of flies with honey and Fe or PQ for 48 hours. (e) Percentage of surviving flies after treatment; (f) locomotor activity test. Results were expressed as percent of control (mean ± SD). Statistical comparisons revealed *P* < 0.05(∗), *P* < 0.01(∗∗), and *P* < 0.001(∗∗∗) between groups.

Since honey induced a prolongation of flies lifespan and the sugar content of honey is only partially related to its protective effect, we tested whether the antioxidant capacity of Brazilian Pampa biome honeys present a role in the protection against oxidative stress* in vivo*. Paraquat (PQ) and iron (Fe) have been widely used to induce oxidative stress in animal models including* Drosophila melanogaster* and are also claimed as chemical-induced models of Parkinsonism [11, 38]. Paraquat (1,1′ dimethyl-4′,4′-bipyridilium dimethylsulphate) is a nonselective herbicide which causes neurotoxicity via oxidative stress-mediated cell death of dopaminergic neurons [11]. Iron (Fe) is an essential metal for many biological processes; however, in excess it might lead to metabolic and neurological impairments associated with movement disorders such as Parkinson's disease [39]. Therefore, Fe and PQ were used to induce oxidative stress in flies, to evaluate whether honey has the ability to reverse oxidative damage.  Figure 4(a) shows that honey, in a cotreatment schedule, fully protected flies against Fe and partially protected against PQ exposure. It was also shown that honey completely blocked Fe and PQ induced locomotor deficits (Figure 4(b)). In order to discard the possibility that the observed protective effect of honey against Fe and PQ is solely related to a chelating effect, a pre- and posttreatment schedule was designed, in which honey and oxidative stressors are not concomitantly administered. In a pretreatment condition, honey was administered to flies during 48 hours; then, honey was removed and Fe and PQ were delivered to the experimental animals. In the posttreatment exposure, flies were first treated with Fe or PQ during 48 h then received a honey solution for a period of 48 h as outlined in Figure 4. The pretreatment of flies with honey completely protected against Fe and PQ induced mortality (Figure 4(c)). A significant protection against Fe and PQ induced locomotor impairments was also observed (Figure 4(d)). It was also evaluated whether honey was able to rescue* D. melanogaster* against Fe/PQ induced mortality and locomotor deficits. As illustrated in Figures 4(e) and 4(f), the posttreatment with honey fully recovers the survivorship of flies exposed to Fe and PQ. Honey was also able to significantly recover locomotor changes induced by Fe and PQ (Figure 4(f)).

**Figure 5:**
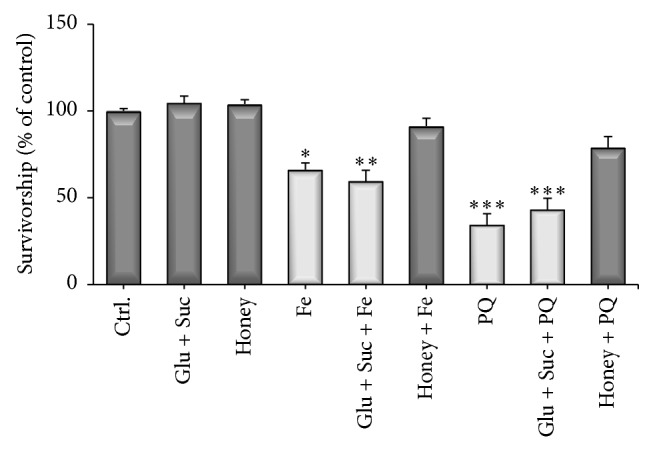
Chronic pretreatment of flies with honey or sugars (7 days) followed by Fe or PQ for 48 hours. After treatments were finished, the number of surviving flies was registered. Results were expressed as percent of control (mean ± SD). Statistical comparisons revealed *P* < 0.05(∗), *P* < 0.01(∗∗), and *P* < 0.001(∗∗∗) between groups.

Honey has about 70–80% of its composition of reducing sugars and 2–6% sucrose, so we asked whether the protection against Fe/PQ could be due to the presence of these sugars, since it was shown previously that reducing sugars can afford some degree of protection in a* Drosophila* model [13]. In order to answer this question, a prolonged treatment (7 days) with the same amount of sugars existing in honey was administered to flies. This would give an insight regarding whether the sugar or nonsugar components of honey are responsible for the observed protection of flies against Fe/PQ toxicity. The results showed that glucose and sucrose equivalents did not protect against Fe/PQ, while in turn honey protected the flies against Fe/PQ toxicity (Figure 5).

It is shown for the first time that Brazilian Pampa biome honeys afford protection against oxidative stress in a* Drosophila* model. It was also shown that the nonsugars components of honey are more likely to participate in this phenomenon. The observed protection can be attributed to the presence of phenolic components in the honey since they are directly related to the antioxidant activity* in vitro* (Table 5). Jimenez-Del-Rio et al. [11] showed that pretreatment with polyphenols might be helpful in reducing iron and paraquat-induced toxicity in* Drosophila*. Our results corroborate with this fact and suggest that the* in vivo* antioxidant effects of honey may be due to the presence of phenols in honey composition. More studies are needed, to identify and isolate these phenolic compounds to determine if the antioxidant activity is due to some specific phenol or due to complex mixture of natural compounds present in honey.

Nevertheless, it is known that Parkinson's disease (PD) is a progressive neurodegenerative disorder characterized by the degeneration of dopaminergic neurons in the substantia nigra pars compacta [38]. The mechanisms that lead to death of dopaminergic neurons remain unclear, but studies have shown a central role for oxidative stress in this process [11]. Thus, current research seeks different neuroprotection strategies, including antioxidant therapies, thus highlighting the potential of natural products in such a context. The protective effect of honey against Fe and PQ observed here provides an initial evidence for the potential use of honey as an adjuvant in the therapeutics of Parkinson's disease.

### 3.5. Total Glucose and Glycogen

**Figure 6:**
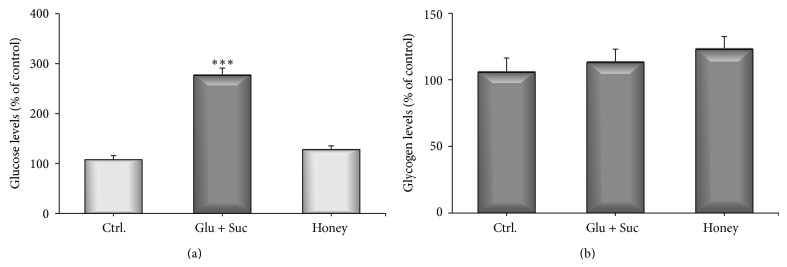
Glucose and glycogen levels of flies treated for 7 days with honey or sugars. (a) Total glucose. (b) Glycogen content. Results were expressed as percent of control (mean ± SD). Statistical comparisons revealed *P* < 0.001(∗∗∗) between groups.

A protective effect of honey against Fe/PQ toxicity after 7 days of treatment was observed (Figure 5). However, as honey has high sugar content, we inquired that maybe this characteristic can produce an increase in glucose levels, characterizing a hyperglycemic effect, which would be detrimental to honey's antioxidant and protective effects in our model. Thus, in order to answer this query, flies were fed during 7 days in honey (10% solution), glucose + sucrose (equivalent amounts as present in honey), and a low sugar control (1% sucrose). No changes on glucose and glycogen levels were observed after 48 and 96 h (data not shown). However, after 7 days of treatment, a significant increase in total glucose was observed on the glucose + sucrose treated group (*P* < 0.0001) when compared to the control and honey diet. Honey diet did not differ from the control, indicating a hypoglycemic-like activity (Figure 6(a)), since honey equivalent amounts of sugars were able to raise glucose levels in flies. The glycogen levels remained constant on all diets tested (Figure 6(b)).

Despite the high sugar content of honey, our results showed that this natural product did not change glucose levels in treated flies, when substituted equivalently by common sugars, even after a prolonged administration schedule. In fact, a hypoglycemic effect of honey has been shown in humans [9], both normal as well as type I diabetes patients, pointing out the beneficial effects of honey also as an antihyperglycemic and antidiabetic agent. Further studies are needed to elucidate the role of honey in the glucose metabolism in* Drosophila* as well as its potential use as a therapeutic agent against metabolic diseases.

## 4. Conclusion

At least in our knowledge, this is the first report on the quality and identity of the Brazilian Pampa biome honey. The results demonstrate its great value in terms of quality parameters, overcoming National and International standards. A prominent antioxidant activity* in vitro* and* in vivo* was also demonstrated, indicating the potential use of this natural product as an alternative supplement on the therapy of important human disease-conditions, such as Parkinson's disease and diabetes. Our study also highlights the use of* Drosophila melanogaster* as a viable model for preliminary studies on the antioxidant aspects of honey and its constituents. Additional studies are needed to assess potential therapeutic properties of honey in the management of chronic diseases associated with oxidative stress.

## Figures and Tables

**Table 1:** Harvest period and floral origin of ten different honeys from Brazilian Pampa biome.

	Harvest	Floral origin
1	October 2012	Multifloral-wildflowers
2	November 2012	Multifloral-wildflowers
3	January 2013	Multifloral-wildflowers
4	January 2013	Multifloral-wildflowers
5	September 2012	Multifloral-bushland
6	January 2013	Multifloral-wildflowers
7	September 2012	Multifloral-bushland
8	March 2012	Multifloral-wildflowers
9	August 2012	Monofloral-*Brassica napus *
10	May 2012	Monofloral-*Eucalyptus grandis *

**Table 2:** Physicochemical analysis of Brazilian Pampa biome honey samples.

	Moisture %	Insoluble solids (%)	pH	Free acidity (mEq/Kg)	Reducing sugars (%)	Apparent sucrose (%)	Total protein (mg/g)	HMF (mg/Kg)	Lugol's reaction	Lund's reaction (mL)
1	20.0 ± 0.0	<0.00	4.3 ± 0.0	39.2 ± 0.9	72.2 ± 0.3	5.9 ± 1.3	10.9 ± 0.2	8.7 ± 1.0	Negative	0.7 ± 0.0
2	18.6 ± 0.0	<0.0	4.0 ± 0.0	44.2 ± 0.4	84.6 ± 1.9	2.3 ± 0.3	12.6 ± 0.5	17.8 ± 2.1	Negative	0.7 ± 0.0
3	19.4 ± 0.0	<0.0	3.9 ± 0.0	32.7 ± 0.1	75.8 ± 2.7	4.3 ± 0.4	15.0 ± 0.4	14.4 ± 0.8	Negative	0.8 ± 0.0
4	19.0 ± 0.0	<0.00	3.9 ± 0.0	33.3 ± 0.9	83.5 ± 0.9	4.9 ± 0.3	15.2 ± 0.5	15.7 ± 0.0	Negative	0.8 ± 0.0
5	19.0 ± 0.0	<0.0	4.3 ± 0.0	25.8 ± 0.0	79.7 ± 2.7	4.2 ± 0.8	8.0 ± 0.4	ND∗	Negative	0.7 ± 0.0
6	18.6 ± 0.0	<0.00	5.0 ± 0.0	13.0 ± 0.6	78.5 ± 2.4	5.3 ± 1.2	7.1 ± 0.6	1.6 ± 0.1	Negative	0.7 ± 0.0
7	19.8 ± 0.0	<0.00	3.7 ± 0.0	24.6 ± 0.5	76.9 ± 0.1	5.1 ± 0.8	10.5 ± 0.8	3.7 ± 0.3	Negative	0.7 ± 0.0
8	19.0 ± 0.0	<0.00	4.3 ± 0.0	26.3 ± 2.1	78.1 ± 2.1	2.9 ± 0.8	10.2 ± 0.2	2.3 ± 0.1	Negative	0.7 ± 0.0
9	18.8 ± 0.0	<0.00	3.9 ± 0.0	22.2 ± 0.4	82.5 ± 0.9	2.3 ± 0.9	7.9 ± 0.4	8.9 ± 0.2	Negative	0.7 ± 0.0
10	18.2 ± 0.0	<0.00	4.4 ± 0.0	32.2 ± 0.6	76.8 ± 0.9	3.0 ± 0.5	11.4 ± 0.7	3.2 ± 0.1	Negative	1.0 ± 0.0

Data are expressed as mean ± SD. ^*^ND: not detected.

**Table 3:** Brazilian legislation requirements for physicochemical parameters.

Parameter	Limits
Moisture (%)	Maximum 20
Insoluble solids (%)	Maximum 0.1
pH	#
Free acidity (mEq/Kg)	Maximum 50
Reducing sugars (%)	Minimum 65
Apparent sucrose (%)	Maximum 6
Total protein (mg/g)	#
HMF (mg/Kg)	Maximum 60
Lugol's reaction	Qualitative-negative
Lund's reaction (mL)	Maximum 0.6–3.0

#: There is no regulation or legislation imposing limits.

**Table 4:** Brazilian Pampa biome honeys *in vitro* antioxidant properties.

	Phenols (mg of GAE^a^/100 g )	Flavonoids (mg of QE^b^/100 g)	FRAP (*μ*M of Fe (II)/100 g)	Percentage scavenging of DPPH	Percentage scavenging of ABTS	ABS_450_ (mAU)
1	64.5 ± 2.0	2.4 ± 0.2	461.3 ± 1.2	37.6 ± 4.2	31.3 ± 2.9	463.0 ± 0.0
2	69.0 ± 5.1	ND	358.0 ± 13.0	33.1 ± 0.5	27.1 ± 0.4	490.0 ± 8.5
3	63.7 ± 2.8	1.8 ± 0.4	318.0 ± 34.2	21.9 ± 3.3	25.5 ± 0.5	432.0 ± 11.3
4	63.5 ± 6.7	2.5 ± 0.3	314.7 ± 8.2	29.5 ± 2.8	24.0 ± 1.7	438.5 ± 16.3
5	41.0 ± 4.3	2.5 ± 0.0	271.3 ± 24.7	22.4 ± 1.3	15.9 ± 5.0	308.5 ± 10.6
6	46.5 ± 6.5	4.2 ± 0.5	290.5 ± 18.9	27.5 ± 0.9	21.0 ± 0.5	209.0 ± 0.0
7	47.0 ± 4.3	2.3 ± 0.5	133.0 ± 5.9	22.3 ± 1.9	14.0 ± 0.2	120.5 ± 4.9
8	56.2 ± 8.3	2.3 ± 0.4	378.8 ± 18.9	35.9 ± 0.2	27.7 ± 0.1	374.0 ± 7.1
9	28.9 ± 1.6	1.8 ± 0.4	145.5 ± 30.6	20.9 ± 2.0	16.5 ± 0.4	188.0 ± 0.0
10	61.9 ± 3.7	3.4 ± 0.5	537.2 ± 4.7	44.2 ± 3.7	34.3 ± 2.8	488.5 ± 19.1

Data are expressed as mean ± SD. ^a^GAE: gallic acid equivalent; ^b^QE: quercetin equivalent. DPPH/ABTS radical scavenging refers to 10% honey solution. ABS_450_ refers to honey color intensity; ∗ND: not detected.

**Table 5:** Correlation analysis of Brazilian Pampa biome honey *in vitro* antioxidant activity. Total phenolics (TP), flavonoids content (FC), and color intensity (ABS_450_).

Parameter^a^	TP	FC	FRAP	DPPH	ABTS	ABS_450_
TP						
FC	0.258					
FRAP	**0.715** ∗	0.147				
DPPH	0.615	0.136	**0.903** ∗			
ABTS	**0.782** ∗	0.001	**0.950** ∗	**0.888** ∗		
ABS_450_	**0.844** ∗	−0.280	**0.844** ∗	**0.675** ∗	**0.862** ∗	

^a^Statistically significant Pearson correlation coefficients are indicated by ∗*P* < 0.05.
